# Supplementary material for: Causal Relationship between Aging and Anorexia Nervosa: A White-Matter-Microstructure-Mediated Mendelian Randomization Analysis
Source: Biomedicines. 2024 Aug 16;12(8):1874. doi: 10.3390/biomedicines12081874 (PMC11351342; doi:10.3390/biomedicines12081874)
Supplement: Supplementary file 1 [file biomedicines-12-01874-s001.zip › Supplementary Figure/Figure S4/Singe SNP plot of MR analysis for TL to ubm-b-1899.pdf]

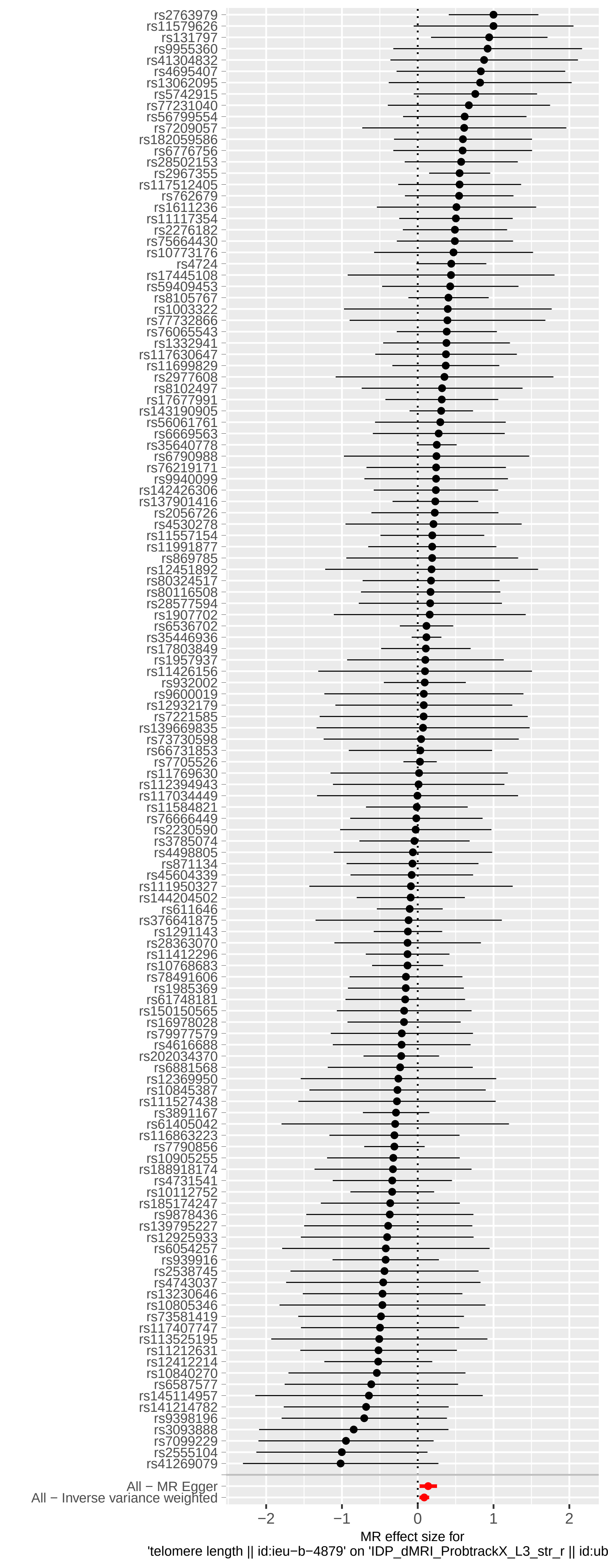
rs2763979
rs11579626
rs131797
rs9955360
rs41304832
rs4695407
rs13062095
rs5742915
rs77231040
rs56799554
rs7209057
rs182059586
rs6776756
rs28502153
rs2967355
rs117512405
rs762679
rs1611236
rs11117354
rs2276182
rs75664430
rs10773176
rs4724
rs17445108
rs59409453
rs8105767
rs1003322
rs77732866
rs76065543
rs1332941
rs117630647
rs11699829
rs2977608
rs8102497
rs17677991
rs143190905
rs56061761
rs6669563
rs35640778
rs6790988
rs76219171
rs9940099
rs142426306
rs137901416
rs2056726
rs4530278
rs11557154
rs11991877
rs869785
rs12451892
rs80324517
rs80116508
rs28577594
rs1907702
rs6536702
rs35446936
rs17803849
rs1957937
rs11426156
rs932002
rs9600019
rs12932179
rs7221585
rs139669835
rs73730598
rs66731853
rs7705526
rs11769630
rs112394943
rs117034449
rs11584821
rs76666449
rs2230590
rs3785074
rs4498805
rs871134
rs45604339
rs111950327
rs144204502
rs611646
rs376641875
rs1291143
rs28363070
rs11412296
rs10768683
rs78491606
rs1985369
rs61748181
rs150150565
rs16978028
rs79977579
rs4616688
rs202034370
rs6881568
rs12369950
rs10845387
rs111527438
rs3891167
rs61405042
rs116863223
rs7790856
rs10905255
rs188918174
rs4731541
rs10112752
rs185174247
rs9878436
rs139795227
rs12925933
rs6054257
rs939916
rs2538745
rs4743037
rs13230646
rs10805346
rs73581419
rs117407747
rs113525195
rs11212631
rs12412214
rs10840270
rs6587577
rs145114957
rs141214782
rs9398196
rs3093888
rs7099229
rs2555104
rs41269079
All − MR Egger
All − Inverse variance weighted
−2
−1
0
1
2
MR effect size for
'telomere length || id:ieu−b−4879' on 'IDP_dMRI_ProbtrackX_L3_str_r || id:ub
